# Characterization of the *Dicranostigma leptopodum* chloroplast genome and comparative analysis within subfamily Papaveroideae

**DOI:** 10.1186/s12864-022-09049-8

**Published:** 2022-12-02

**Authors:** Lei Wang, Fuxing Li, Ning Wang, Yongwei Gao, Kangjia Liu, Gangmin Zhang, Jiahui Sun

**Affiliations:** 1 College of Horticulture and Plant Protection, Henan University of Science and Technology, Luoyang, 471023 Henan China; 2 Laboratory of Systematic Evolution and Biogeography of Woody Plants, School of Ecology and Nature Conservation, Beijing Forestry University, Beijing, 100083 China; 3 State Key Laboratory Breeding Base of Dao‑di Herbs, National Resource Center for Chinese Materia Medica, China Academy of Chinese Medical Sciences, Beijing, 100700 China

**Keywords:** Chloroplast genome, *Dicranostigma leptopodum*, Papaveroideae, Phylogeny, Genetic diversity

## Abstract

**Background:**

*Dicranostigma leptopodum* (Maxim.) Fedde is a perennial herb with bright yellow flowers, well known as "Hongmao Cao" for its medicinal properties, and is an excellent early spring flower used in urban greening. However, its molecular genomic information remains largely unknown. Here, we sequenced and analyzed the chloroplast genome of *D. leptopodum* to discover its genome structure, organization, and phylogenomic position within the subfamily Papaveroideae.

**Results:**

The chloroplast genome size of *D. leptopodum* was 162,942 bp, and *D. leptopodum* exhibited a characteristic circular quadripartite structure, with a large single-copy (LSC) region (87,565 bp), a small single-copy (SSC) region (18,759 bp) and a pair of inverted repeat (IR) regions (28,309 bp). The *D. leptopodum* chloroplast genome encoded 113 genes, including 79 protein-coding genes, 30 tRNA genes, and four rRNA genes. The dynamics of the genome structures, genes, IR contraction and expansion, long repeats, and single sequence repeats exhibited similarities, with slight differences observed among the eight Papaveroideae species. In addition, seven interspace regions and three coding genes displayed highly variable divergence, signifying their potential to serve as molecular markers for phylogenetic and species identification studies. Molecular evolution analyses indicated that most of the genes were undergoing purifying selection. Phylogenetic analyses revealed that *D. leptopodum* formed a clade with the tribe Chelidonieae.

**Conclusions:**

Our study provides detailed information on the *D. leptopodum* chloroplast genome, expanding the available genomic resources that may be used for future evolution and genetic diversity studies.

**Supplementary Information:**

The online version contains supplementary material available at 10.1186/s12864-022-09049-8.

## Introduction

**Fig. 1:**
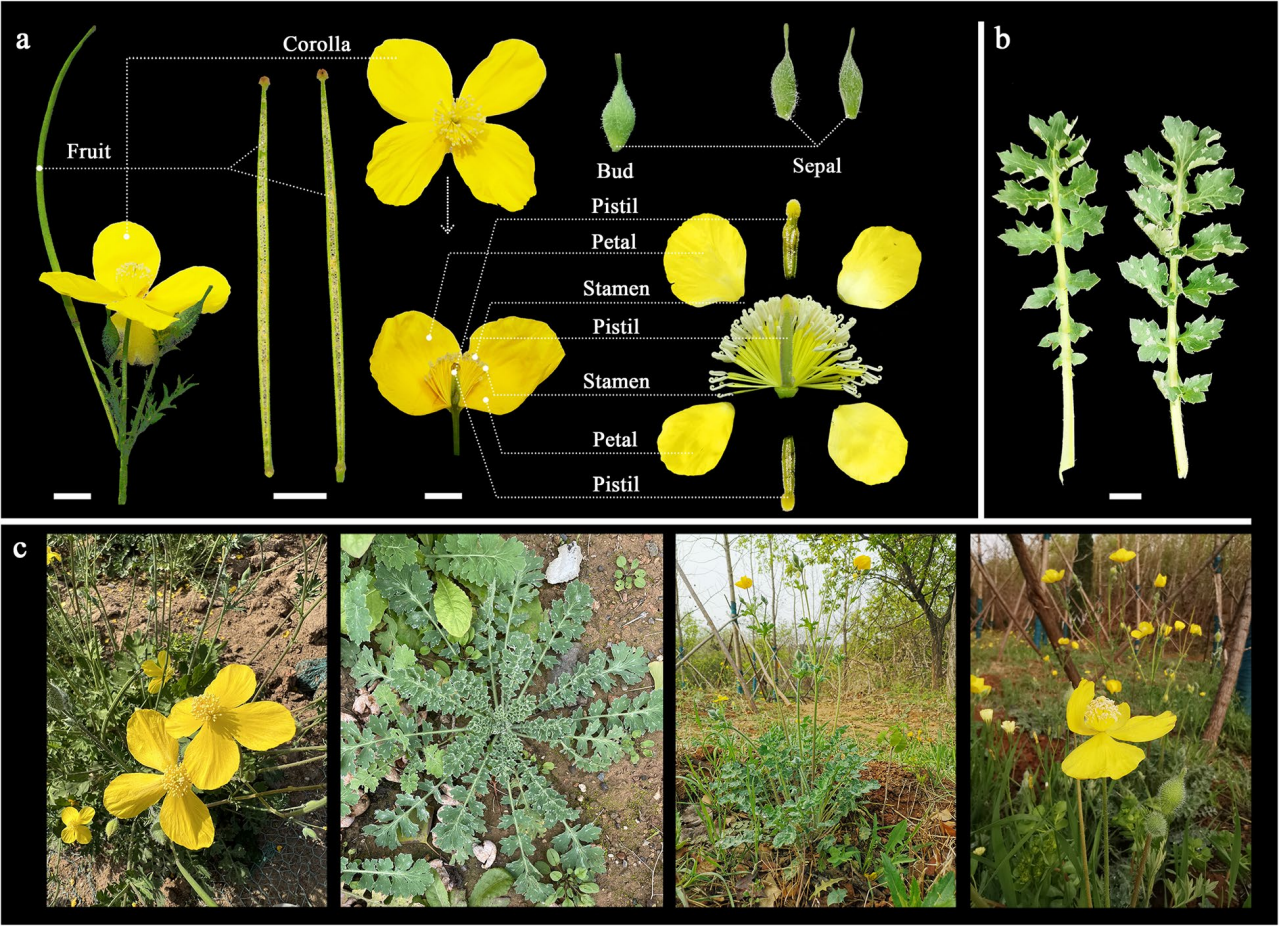
Floral anatomy of *D. leptopodum* and its habitat. **a**. Floral anatomy of *D. leptopodum*; **b**. leaf morphology of *D. leptopodum*; c. morphology and habitat of the whole plant of *D. leptopodum*; scale bars, 1.0 cm

*Dicranostigma leptopodum* (Maxim.) Fedde within Papaveraceae is a perennial herb with bright yellow flowers that is endemic to China and is well known as "Hongmao Cao" due to its medicinal properties [1–3]. The distribution of the species ranges from southwest and northwest to central China, and its fluorescence occurs from March to May, lasting for one or two months (Fig. 1). The basal leaves of *D. leptopodum* are dense and may be evergreen throughout the winter, with a capacity to retain moisture and fertilizer in soil [4, 5]. Some studies have shown that *D. leptopodum* has a certain tolerance for and ability to enrich elements such as Cd, Zn, Cu, and Pb and can be used as a plant for remediating heavy-metal-contaminated soils in mining areas, farmland, and rivers [6]. Therefore, from the perspectives of both ornamental characteristics and ecological restoration, *D. leptopodum* and its related species are rare, excellent early spring flowering resources for urban greening [7].

The phylogenetic position of *Dicranostigma* remains unclear. Some classical taxonomic studies suggest that *Dicranostigma* and *Glaucium* are sister groups within Papaveraceae [8]; however, the latest molecular evidence shows that *Dicranostigma* and *Glaucium* are still sister groups but within Chelidonieae [9], which is inconsistent with some previous findings based on morphological evidence [10]. Thus, the phylogenetic position of *Dicranostigma* remains controversial.

The chloroplast genome, which is characterized primarily by maternal inheritance, has been widely used in the reconstruction of phylogenetic relationships at different taxonomic levels [11–15] and species identification [16] due to its highly conserved nature in terms of stable structure, gene arrangement, GC (guanine and cytosine) content, and lack of recombination during genetic processes. To date, most of the studies on *Dicranostigma* have focused mainly on the medicinal properties of its extracts [1, 2], ecophysiological index, and seed germination characteristics [5]; in contrast, almost no research has been conducted on its phylogeny. Additionally, only a few molecular sequences of species within *Dicranostigma* are recorded in the NCBI database, substantially restricting phylogenetic research and the development and utilization of *D. leptopodum*.

Therefore, this study intends to comprehensively reveal the phylogeny of *D. leptopodum* and its relatives using comparative chloroplast genomic and molecular phylogenetic methods. The main research objectives are as follows: (1) to comprehensively analyze the structural characteristics of the *D. leptopodum* chloroplast genome, (2) to identify similarities and differences in the structural characteristics of the chloroplast genome of *D. leptopodum* and its relatives, and (3) to reveal the phylogenetic position of *Dicranostigma* within Papaveraceae.

## Materials and methods

### Plant material, DNA extraction, and genome sequencing

The *D. leptopodum* material was obtained from Luoyang, Henan, China. The specimens were subsequently deposited in the Herbarium of the College of Horticulture and Plant Protection, Henan University of Science and Technology (Voucher: WL1000). These specimens were identified by Dr. Ning Wang at Henan University of Science and Technology. Fresh leaves were preserved in silica gel. Total DNA was extracted using a modified CTAB method [17] and detected by electrophoresis on 0.8% agarose gels. Next, library preparation and next-generation sequencing (Illumina, Nova PE150 sequencing strategy) were conducted at Novogene Biotechnology Co., Ltd. (Tianjin, China). The total sequencing data were 4 Gb.

### Chloroplast genome assembly and annotation

Trimmomatic v 0.39 [18] was used to filter the original reads obtained by sequencing and remove those of low quality at the joints and ends. The chloroplast genome was assembled using GetOrganelle v 1.7.6.1 [19] with the default parameters. Plastid Genome Annotator (PGA) software [20] was used to annotate the chloroplast genome with the reference of *Coreanomecon hylomeconoides* Nakai (KT274030). Furthermore, the results annotated by PGA were checked using Geneious Prime v 2021 [21] and Sequin v 9.20 [22] to ensure the accuracy of the annotation results. Some genes with high sequence divergence and genes with introns received special attention, such as *accD*, *petB*, *petD*, *rps16*, *rpl16*, and *ycf1*. We manually checked the annotations of these genes. The chloroplast genome map of *D. leptopodum* was drawn and visualized using OGDRAW v 1.3.1 [23]. The complete annotated sequence was submitted to GenBank with accession number OM994890. Codon usage and relative synonymous codon usage (RSCU) analyses were performed using the MEGA-X program [24] to analyze the codon usage bias of the *D. leptopodum* chloroplast genome.

### Comparative analyses of chloroplast genome structures

We compared the chloroplast genome structures within species of the subfamily Papaveroideae. Eight samples of *Dicranostigma leptopodum* (OM994890)*, Stylophorum lasiocarpum* (Oliv.) Fedde (MW232434), *Papaver nudicaule* L. (MW411801)*, Meconopsis horridula* Hook. f. & Thomson (MK533646)*, Macleaya cordata* (Willd.) R. Br. (MT178411)*, Hylomecon japonica* (Thunb.) Prantl & Kündig (MK251463)*, Coreanomecon hylomeconoides* Nakai (KT274030)*,* and *Chelidonium majus* L. (MK433200) were used. The mVISTA online program (https://genome.lbl.gov/vista/mvista/submit.shtml) was used to compare the genome structures and the sequence similarity of the chloroplast genomes of eight Papaveroideae species. The annotated *D. leptopodum* chloroplast genome was used as a reference. The variation in LSC/IRb/SSC/IRa junction regions was compared using IRscope [25] (https://irscope.shinyapps.io/IRapp/).

Repeat sequences were analyzed using the REPuter online program [26] and Perl script MISA [27]. REPuter was used to identify four types of long repeats (forward, reverse, palindromic, and complementary) in eight Papaveroideae chloroplast genomes with a Hamming distance of 3 and a minimum repeat size of 30 bp. MISA was employed to identify the simple sequence repeats (SSRs or microsatellites). Six types of SSRs (mono-, di-, tri-, tetra-, penta-, and hexanucleotides) were analyzed, and the minimum numbers of SSRs were set to 10, 5, 4, 3, 3, and 3, respectively.

### Analysis of the nucleotide substitution rate of protein-coding genes

The molecular evolution between *D. leptopodum* and seven other Papaveroideae species was investigated. The values of dN (nonsynonymous substitutions), dS (synonymous substitutions), and ω (dN/dS ratio) were estimated using the YN100 module in PAMLX [28]. All 79 protein-coding genes were extracted from the annotated *D. leptopodum* chloroplast genome. Three strategies were used to infer the molecular evolution: (1) calculate each coding gene for all of the species in a pairwise manner; (2) calculate gene groups with the same function [29], such as *rps*, *pet*, and *psa*; and (3) calculate groups at the species level by combining all 79 coding genes for each species.

### Phylogenetic inference

The newly assembled chloroplast genome of *D. leptopodum* and all the published complete chloroplast genomes of the family Papaveraceae were downloaded from GenBank [22]. All 79 protein-coding genes and the four rRNA genes were extracted from the results of the chloroplast genome annotation. All genes were aligned using MAFFT v 7.450 [30].

Maximum likelihood (ML) and Bayesian inference (BI) methods were used to infer the phylogenetic relationships. The ML tree was constructed using RAxML-NG [31] with the GTR + G model, and node support was assessed with 1000 bootstrap inferences. The BI tree was inferred with MrBayes v 3.2 [32]. The Markov chain Monte Carlo algorithm was performed with two independent runs of four simultaneous chains with a random starting tree and default priors for 5,000,000 generations, with every 1,000 generations used for tree sampling. Tracer v1.6 was used to analyze the convergence to stationary distribution and the effective size of each parameter. The first 25% of generations were discarded as a burn-in. The remaining trees were used to build the BI tree of posterior probabilities.

## Results

### Assembly and general features of the *D. leptopodum* chloroplast genome

**Fig. 2:**
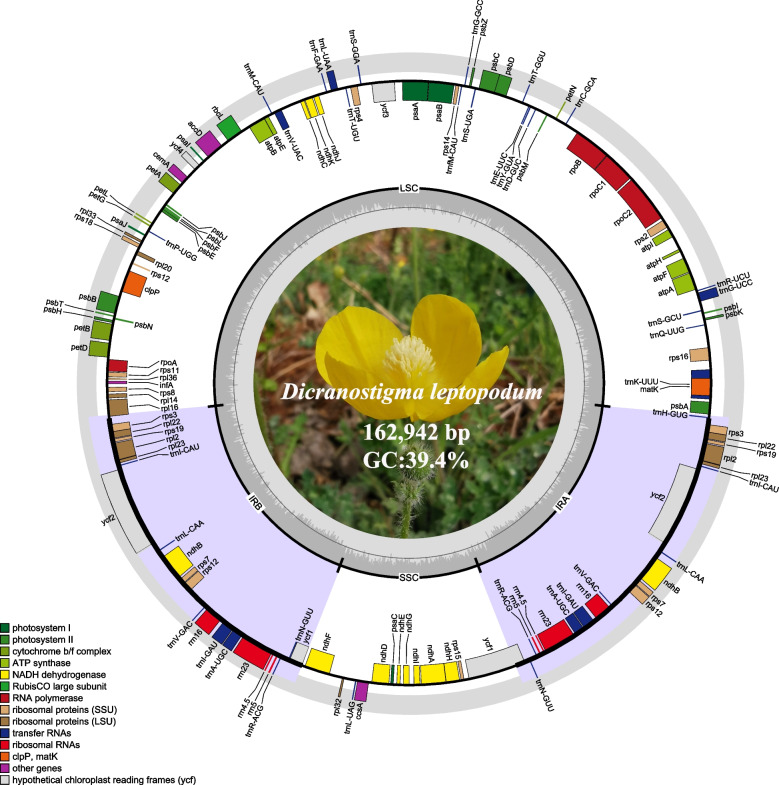
Gene map of the *D. leptopodum* chloroplast genome. Genes located outside the circle are transcribed counterclockwise, while genes inside the circle are transcribed clockwise. In the inner circle, the dark gray area represents the GC content of the cp genome, and the light gray area represents the AT content. Different colored blocks represent genes from different functional groups

Using the Illumina sequencing platform, we obtained 6,536,061 clean reads. The coverage depth of the *D. leptopodum* chloroplast genome was 40 X. The complete *D. leptopodum* chloroplast genome was 162,942 bp, exhibiting a characteristic circular quadripartite structure. The *D. leptopodum* chloroplast genome consisted of a pair of inverted repeat (IR) regions (28,309 bp) separated by a larger single-copy (LSC) region (87,565 bp) and a small single-copy (SSC) region (18,759 bp). A circular map of the chloroplast genome is shown in Fig. 2.

One hundred thirteen unique gene annotations were identified in the *D. leptopodum* chloroplast genome, including 79 protein-coding genes, 30 tRNAs, and four rRNAs. Ten protein-coding genes, four rRNAs, and seven tRNAs were duplicated in the IR regions. The *ycf1* in IRb is a pseudogene formed due to the expansion of the IR region (Fig. 2). Forty-five of those genes play a role in photosynthesis, and 60 genes are associated with self-replication (Table 1). Ten protein-coding genes (*atpF*, *ndhA*, *ndhB*, *petB*, *petD*, *rpl2*, *rpl16*, *rpoC1*, *rps16*, and *rps12*) and six tRNA genes (*trnA-UGC*, *trnG*-*UCC*, *trnI*-*GAU*, *trnK*-*UUU*, *trnL*-*UAA*, and *trnV*-*UAC*) contained a single intron, whereas two genes (*clpP* and *ycf3*) contained two introns. The largest intron was observed in *trnK*-*UUU* (2,558 bp), which contains the *matK* gene. *rps12* is a trans-spliced gene with the 5' end located at the LSC region and the 3' end located in the IR region. The GC content of the complete chloroplast genome was 39.4%, with a higher GC content in the IR region (43.1%) than in the LSC (38.1%) and SSC (34.4%) regions.

**Table 1:** Genes in the *D. leptopodum* chloroplast genome

Category of Genes	Group of Genes	Name of Genes
	Ribosomal proteins (LSU)	*rpl*2, *rpl*14, *rpl*16, *rpl*20, *rpl*22, *rpl*23, *rpl*32, *rpl*33, *rpl*36
	Ribosomal proteins (SSU)	*rps*2, *rps*3, *rps*4, *rps*7, *rps*8, *rps*11, *rps*12, *rps*14, *rps*15, *rps*16, *rps*18, *rps*19
	RNA polymerase	*rpo*A, *rpo*B, *rpo*C1, *rpo*C2
	Ribosomal RNAs	*rrn*16, *rrn*23, *rrn*4.5, *rrn*5
Self-replication	Transfer RNAs	*trn*A-UGC, *trn*C-GCA, *trn*D-GUC, *trn*E-UUC, *trn*F-GAA, *trn*G-GCC,
		*trn*G-UCC, *trn*H-GUG, *trn*I-CAU, *trn*I-GAU, *trn*K-UUU, *trn*L-CAA,
		*trn*L-UAA, *trn*L-UAG, *trn*M-CAU, *trnf*M-CAU, *trn*N-GUU, *trn*P-UGG,
		*trn*Q-UUG, *trn*R-ACG, *trn*R-UCU, *trn*S-GCU, *trn*S-GGA, *trn*S-UGA,
		*trn*T-GGU, *trn*T-UGU, *trn*V-GAC, *tr*nV-UAC, *trn*W-CCA, *trn*Y-GUA
	Translational initiation factor	*inf*A
	ATP synthase	*atp*A, *atp*B, *atp*E, *atp*F, *atp*H, *atp*I
	Protease	*clp*P1
	RubisCO large subunit	*rbc*L
Genes for photosynthesis	NADH dehydrogenase	*ndh*A, *ndh*B, *ndh*C, *ndh*D, *ndh*E, *ndh*F, *ndh*G, *ndh*H, *ndh*I, *ndh*J, *ndh*K
	Cytochrome b/f complex	*pet*A, *pet*B, *pet*D, *pet*G, *pet*L, *pet*N
	Photosystem I	*psa*A, *psa*B, *psa*C, *psa*I, *psa*J
	Photosystem II	*psb*A, *psb*B, *psb*C, *psb*D, *psb*E, *psb*F, *psb*H, *psb*I, *psb*J, *psb*K, *psb*L, *psbM*, *psbN*, *psb*T, *psb*Z
	Subunit of acetyl-CoA carboxylase	*acc*D
Other genes	Maturase	*mat*K
	C-type cytochrome synthesis gene	*ccs*A
	Envelope membrane protein	*cem*A
Genes of unknown function	Conserved open reading frames	*ycf*1, *ycf*2, *ycf*3, *ycf*4

### Codon usage analysis

**Fig. 3:**
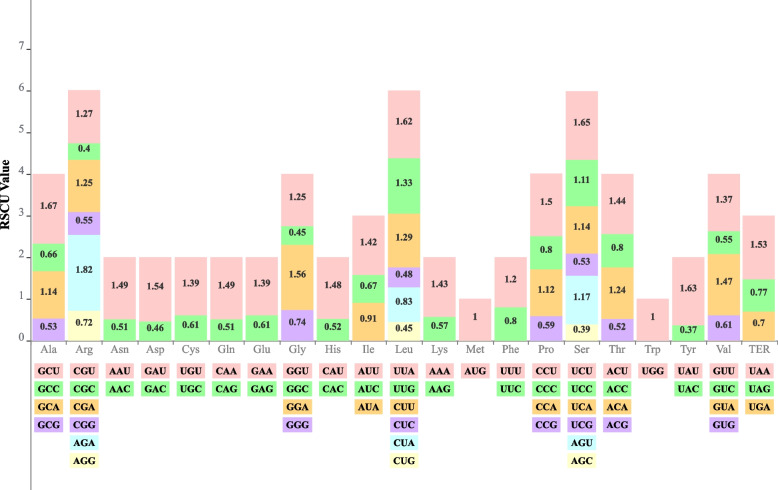
The RSCU values of the 20 amino acids and stop codons of the *D. leptopodum* chloroplast genome and their different codon usages. The color of the histogram corresponds to the color of codons

The *D. leptopodum* chloroplast genome encoded 26,915 codons, representing 20 amino acids and the stop codon (Fig. 3). Leucine was the most abundant amino acid, with a frequency of 10.30%, followed by isoleucine (8.12%) and serine (7.62%); tryptophan was less abundant, with a frequency of 1.81%. All 64 codon types were detected. Additionally, AUU showed a high number of occurrences (1,036), followed by AAA (996), GAA (996), and GAU (953).

We detected 31 codons with a relative synonymous codon usage (RSCU) value > 1, indicating their usage bias in the *D. leptopodum* chloroplast genome. The usage bias was toward A or T (U), with high RSCU values. The highest RSCU values were detected for AGA encoding arginine, followed by GCU encoding alanine and UAU encoding tyrosine. The start codon (ATG) and TGG, encoding tryptophan, exhibited no bias (RSCU = 1).

### Repeat sequences and SSR analysis

**Fig. 4:**
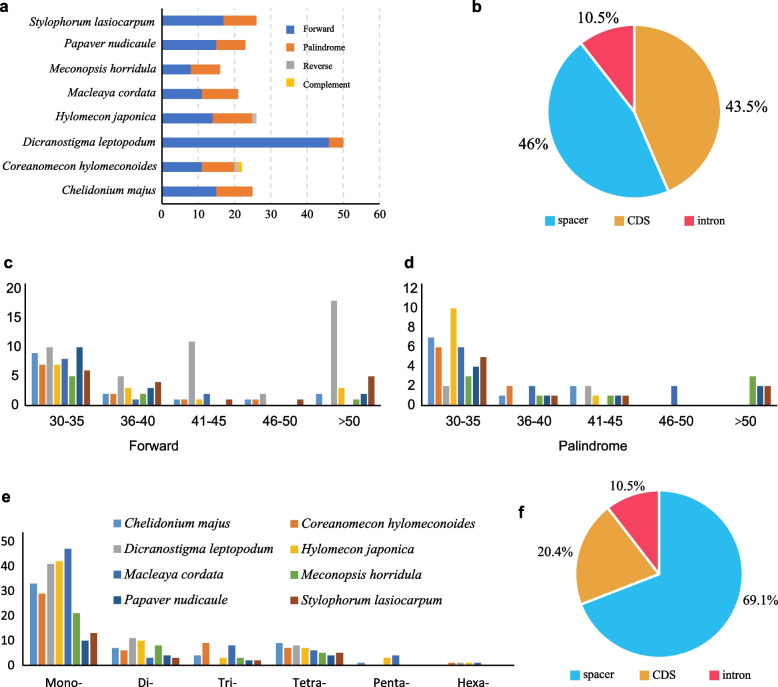
Number and type of long repeats and SSRs in the eight Papaveroideae chloroplast genomes. **a** Number and type of long repeats; **b** frequency of long repeats in LSC, IR, and SSC regions; **c** length of forward long repeats; **d** length of palindromic long repeats; e: number and type of SSRs; and f: frequency of identified SSRs in LSC, IR, and SSC regions

The repeat sequences were investigated among eight Papaveroideae species. Long repeat sequences with a repeat unit longer than 30 bp were analyzed. A total of 210 long repeats (30–190 bp) were identified from the eight chloroplast genomes, consisting of 137 forward, 69 palindromic, two reverse, and one complementary repeat (Fig. 4a). For each chloroplast genome, the number of long repeats ranged from 16 (*Meconopsis horridula*) to 50 (*D. leptopodum*), and the numbers of forward and palindromic repeats were 8–46 and 4–11 (Figs. 4c and 4d), respectively. The two reverse repeats occurred in *Coreanomecon hylomeconoides* and *Hylomecon japonica*, and the only complementary repeat was detected in *Co*. *hylomeconoides*. Most long repeats were distributed in the noncoding areas (including intergenic and intron regions), and a few existed in shared genes, such as *accD*, *ycf1*, and *ycf2* (Fig. 4b and Table S1). The most frequent repeat size was 30–35 bp (51.92%) (Figs. 4c and 4d), and the longest repeat occurred in the *rbcL-accD* sequence of *D. leptopodum*.

The total number of SSRs in the eight chloroplast genomes was 382 (Fig. 4e), ranging from 20 (*Papaver nudicaule*) to 69 (*Macleaya cordata*). Most of the SSRs, with proportions from 55.0% (*Chelidonium majus*) to 74.2% (*Stylophorum lasiocarpum*) of the total number of SSRs, were distributed in the spacer regions. Mononucleotide repeat units were the most frequent type, and A/T repeat units were the most abundant repeats. AT/TA and ATT/TTA repeats were the most frequent repeat units for the dinucleotide and trinucleotide types. We found that the chloroplast genome SSRs in Papaveroideae species contained a high level of AT repeats. The SSRs were mainly located in the noncoding regions (79.58%) (Fig. 4f). We also detected SSRs in protein-coding gene regions, such as *rpoC2*, *ycf1*, and *cemA* (Table S2).

### Analysis of the chloroplast genome structure

We compared the chloroplast genome of *D. leptopodum* to the genomes of seven other species from Papaveroideae. Our comparison indicated that all eight species had similar genome structures and encoded a consistent gene content (Table 2). All eight chloroplast genomes displayed a typical quadripartite structure, and the genome size ranged from 152,867 bp (*P*. *nudicaule*) to 163,107 bp (*Ma*. *cordata*). The LSC regions ranged from 83,104–88,165 bp, and the SSC size was 17,898–18,759 bp. The GC content of the eight chloroplast genomes was very similar (38.7–39.4%) but differed by region. Specifically, the GC content in the IR regions (42.8–43.2%) was higher than the GC content in the LSC (37.2–38.1%) and SSC (32.8–34.4%) regions. The Papaveroideae chloroplast genomes contained highly similar gene contents, consisting of 79 protein-coding genes, 30 tRNAs, and four rRNAs.

**Table 2:** Features of the chloroplast genomes of *D. leptopodum* and seven Papaveroideae species

Species	*Dicranostigma leptopodum*	*Macleaya cordata*	*Stylophorum lasiocarpum*	*Papaver nudicaule*	*Meconopsis horridula*	*Hylomecon japonica*	*Coreanomecon hylomeconoides*	*Chelidonium majus*
Length (bp)	162,942	163,107	153,196	152,867	153,785	160,011	158,824	159,741
LSC (bp)	87,565	87,919	83,230	83,104	83,899	88,165	86,916	87,697
SSC (bp)	18,759	18,582	18,388	18,301	17,898	18,378	18,538	18,574
IR (bp)	28,309	28,303	25,789	25,731	25,994	26,734	26,685	26,735
LSC (GC%)	38.1	37.0	37.4	37.4	37.2	37.4	32.8	37.2
SSC (GC%)	34.4	33.1	34.0	33.5	33.2	33.2	37.3	33.3
IR (GC%)	43.1	42.8	43.2	43.2	43.1	43.2	43.2	43.1
GC (Total%)	39.4	38.6	38.9	38.9	38.8	38.8	38.7	38.7
Genes	113	113	113	113	113	113	113	113
Coding genes	79	79	79	79	79	79	79	79
tRNA	30	30	30	30	30	30	30	30
rRNA	4	4	4	4	4	4	4	4

**Fig. 5:**
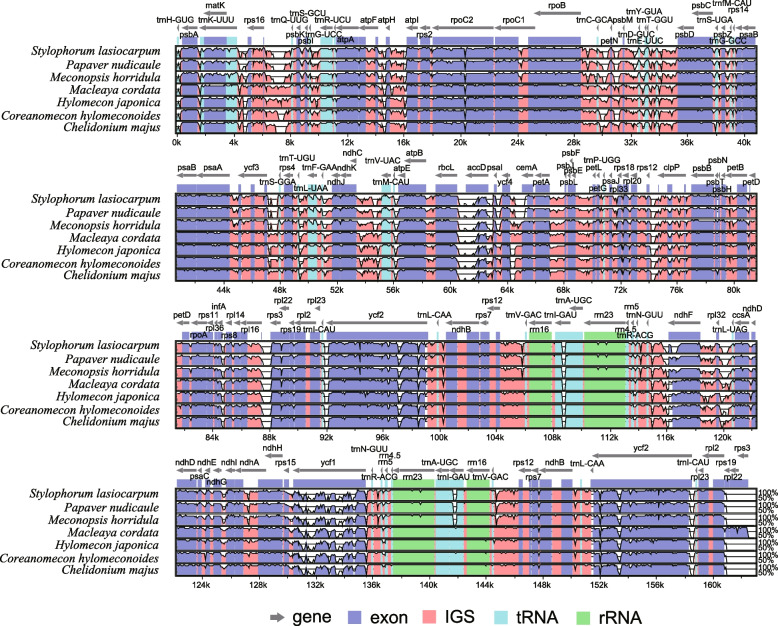
Sequence similarity plot among the eight Papaveroideae chloroplast genomes created using mVISTA. On the y-axis, the percentage of sequence identity is shown between 50 and 100%. The x-axis represents the coordinates in the chloroplast genome. Genome regions are color-coded as protein-coding (exon), tRNAs or rRNAs, and intergenic regions. Genes are signified by gray arrows

Multiple sequence alignments revealed no genomic rearrangements or large inversions in the eight Papaveroideae chloroplast genomes (Fig. 5). These chloroplast genomes were highly conserved both in gene order and identity. Notably, the coding and IR regions were more conserved than noncoding and single-copy regions. The intergenic spacer regions with more variation were *rps16*-*trnQ*, *trnC*-*petN*, *rbcL*-*accD*, *ycf4*-*cemA*, *rps12*-*clpP*, *rpl16*-*trnS*, and *rpl32*-*trnL*. Moreover, the coding genes *accD*, *ndhF* and *ycf1* showed high levels of variation among the coding regions.

**Fig. 6:**
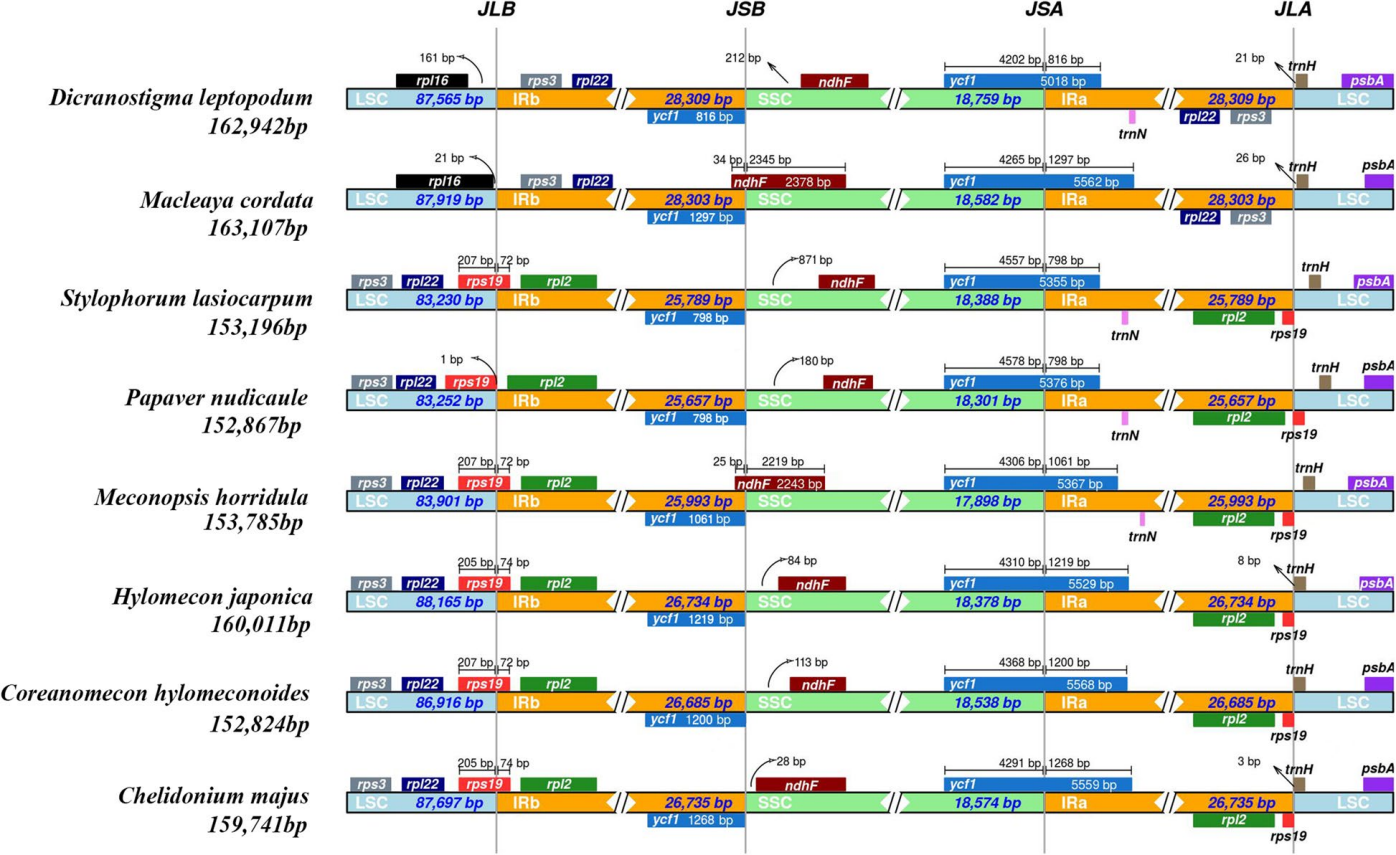
Comparison of junctions between LSC, SSC, and IR regions among eight Papaveroideae species. The distance in the figure is not to scale

Multiple sequence alignment using mVISTA showed that the IR regions of the Papaveroideae chloroplast genomes were highly conserved, and variations in structure were observed in SC/IR boundary regions (Fig. 6). Among the eight chloroplast genomes, two types were identified in the SC/IR boundary. *D. leptopodum* and *Ma. cordata* had similar structures: *rpl16* was located at the boundaries of the LSC and IRb regions in these two species. In the other six species, these boundaries instead contained the *rps19* gene. This result indicated that expansion of the IR caused a duplication of *rps3*, *rpl22,* and *rps19* in *D. leptopodum* and *Ma. cordata* chloroplast genomes. One base pair was identified between the *rps19* and LSC/IRb boundaries in *P*. *nudicaule*. *Rps19* expanded into the IRb regions in five species, namely, *S*. *lasiocarpum*, *M*. *horridula, H*. *japonica, Co*. *hylomeconoides,* and *Ch*. *majus*, by 72, 72, 74, 72, and 74 bp, respectively. The *ycf1* gene crossed the SSC/IRa boundary in all species, and the length of *ycf1* in the IRa region varied among the eight Papaveroideae species from 798 bp to 1,297 bp, which indicated dynamic variation in the SSC/IRa boundaries. Notable differences were observed among species in the IRa/LSC boundary. The *rps3* and *trnH* genes were located at this boundary in *D. leptopodum* and *Ma. cordata*, which instead contained the *rps19* and *trnH* genes in *S*. *lasiocarpum*, *Me*. *Horridula*, *H*. *japonica*, *Co*. *hylomeconoides*, and *Ch*. *majus*. *P*. *nudicaule* was the only species in which the *rps19* gene within the LSC was detected at the IRa/LSC boundary*.*

### Analysis of selection pressure

**Fig. 7:**
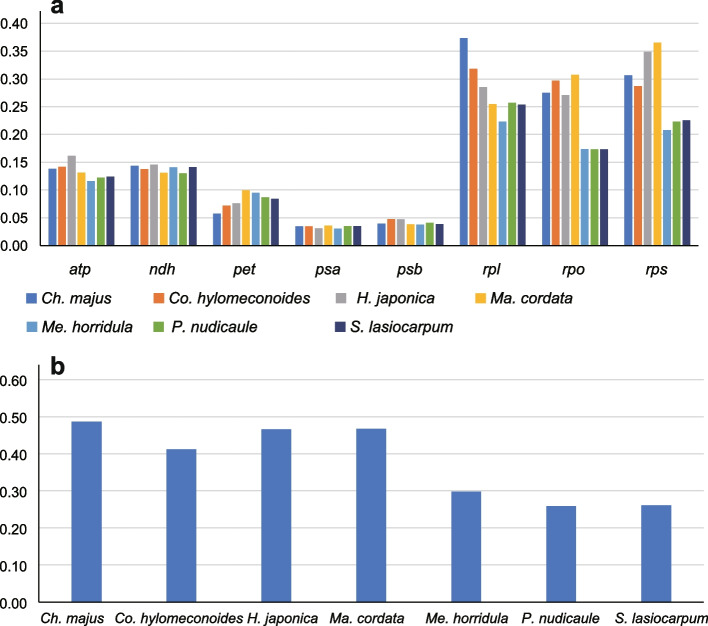
The *dN/dS* values of protein-coding genes in the seven comparative combinations. *D. leptopodum* was used as the reference. a: Gene groups and b: the combination of all 79 protein-coding genes. *dN* nonsynonymous substitution; *dS*, synonymous substitution

The ω values were calculated for 79 single protein genes, gene groups, and combinations of all 79 coding genes for each of the eight Papaveroideae species, with *D. leptopodum* serving as the reference (Table S3 and Fig. 7). Among the 79 single protein-coding genes, most of the ω values were less than one, except for *rpl20* in *Ch*. *majus* (1.3599) and *Co*. *hylomeconoides* (1.2238); *rps7* in *Ch*. *majus* (3.4119), *Co*. *hylomeconoides* (3.4119), *H. japonica* (3.4119), *Ma*. *cordata* (3.4119), and *Me*. *Horridula* (1.242); and *ycf2* in *Ch*. *majus* (1.444), *Co*. *hylomeconoides* (1.4265), *H. japonica* (1.2824), and *Ma*. *cordata* (1.3469). The ω values of all eight gene groups were less than 0.5 (Fig. 7), indicating the presence of purifying selection pressure on the gene groups of Papaveroideae species. At the species level, the ω values among the eight species were not significantly different, suggesting uniform evolution rates in the Papaveroideae species.

### Phylogenetic inference of the family Papaveraceae

**Fig. 8:**
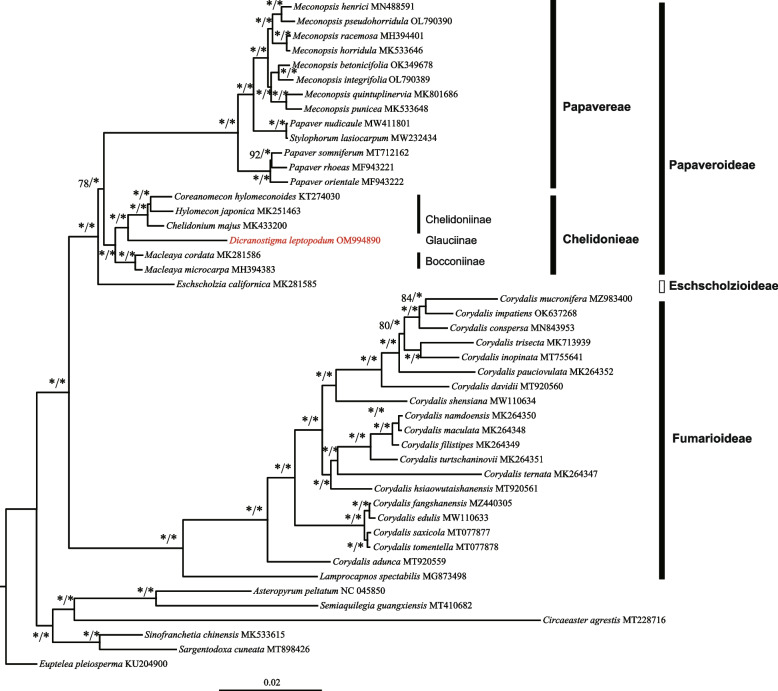
Phylogenetic tree based on 83 chloroplast gene sequences using ML and BI methods. The number above the lines indicates ML bootstrap support (BS) values and Bayesian PP for each node (100 BS or 1.0 PP were marked with "*")

ML and BI trees were constructed based on 83 chloroplast genes to infer the phylogenetic position of *Dicranostigma* in the family Papaveraceae (Fig. 8). This dataset included 20 species of the subfamily Fumarioideae, 19 of the subfamily Papaveroideae, one species (*Eschscholzia californica*) of the subfamily Eschscholzioideae and six species (*Asteropyrum peltatum, Semiaquilegia guangxiensis, Circaeaster agrestis, Sinofranchetia chinensis, Sargentodoxa cuneata,* and *Euptelea pleiosperma*) as the outgroups. Topological structures generated from the ML and BI analyses were consistent and presented highly resolved phylogenies. Papaveraceae was divided into two clades with strong support (bootstrap (BS) value = 100% for the ML tree and posterior probability (PP) = 1.00 for the BI tree), and subfamilies Papaveroideae and Eschscholzioideae formed a clade.

The subfamily Papaveroideae was shaped into two clades, including the tribe Papavereae and the tribe Chelidonieae, both with strong support (BS/PP = 100/1). Our results showed that species of *Papaver* did not form a clade. *P*. *nudicaule* was sister to *S*. *lasiocarpum* and was not clustered in a clade with other *Papaver* species. The tribe Chelidonieae was further divided into three groups with strong support, each belonging to a different subtribe. The first group contained two *Macleaya* species, which belong to the subtribe Bocconiinae. The second group contained *Dicranostigma leptopodum,* which was clustered with the subtribe Chelidoniinae that contained three genera.

## Discussion

In this study, we sequenced the chloroplast genome of *D. leptopodum* and applied it in comparative analyses with the available chloroplast genomes of the subfamily Papaveroideae. The complete chloroplast genome of *D. leptopodum* showed a typical quadripartite structure—with one LSC and one SSC region, as well as two IR regions—which was highly conserved, similar to the chloroplast genomes previously reported in the subfamily Papaveroideae [33, 34]. The genome size of *D. leptopodum* did not differ substantially from the available chloroplast genomes of Papaveroideae. However, the IR size of *D. leptopodum* was much larger than the IR sizes of the other species, except *Ma*. *cordata* (Table 2). The contractions and expansions at the borders of the IR regions are considered the most effective processes causing genome size variations (Fig. 6); furthermore, these contractions and expansions are important evolutionary events in the chloroplast genome, with effects on genome size. After comparing eight chloroplast genomes from Papaveroideae species, we noticed that the boundaries between the LSC and IRb regions were divided into two types (Fig. 6). The IR regions of *D. leptopodum* and *Ma*. *cordata* exhibited an obvious expansion, and those genomes had the largest IR sizes. More genes had changed from a single copy to two copies. We also evaluated the IRa and LSC junctions, and the distributions and locations of genes in these regions were highly variable. Therefore, changes in the SC/IR boundaries may be the main contributors to genome size variation, especially in the IR regions, in Papaveroideae species.

Similar to typical angiosperm chloroplast genomes [35, 36], *D. leptopodum* shared a high sequence similarity with the other Papaveroideae species. However, some regions of these chloroplast genomes exhibited high sequence divergence. According to the mVISTA results, the sequence divergence of the IR region was lower than the sequence divergences of the LSC and SSC regions (Fig. 5), due to the sequence correction of the two copies in the processes of gene replication and transcription. The other reason for the conservation of the IR regions is that they play an important role in maintaining the conservation and stability of the chloroplast genome structure [37, 38]. We identified several intergenic regions and genes with high sequence divergences, such as *rps16*-*trnQ*, *trnC*-*petN*, *rbcL*-*accD*, *ycf4*-*cemA*, *rps12*-*clpP*, *rpl16*-*trnS*, *rpl32*-*trnL*, *accD*, *ndhF*, and *ycf1* (Fig. 5). These high sequence divergence regions have previously been reported in other lineages. For example, Dong et al. [39, 40] compared several pairs of chloroplast genome sequences and identified *rps16*-*trnQ*, *trnC*-*petN*, *rbcL*-*accD*, *rpl32*-*trnL*, *ndhF*, and *ycf1* as mutation hotspot regions that might be used as phylogenetic and species identification markers for angiosperms. Furthermore, these divergent regions are potentially useful genomic markers in phylogenetic studies of the subfamily Papaveroideae. Our results also support the previous report that the LSC region had more highly divergent sequences than the IR and SSC regions, suggesting that LSC regions are evolving rapidly [12].

Repeat sequences play important roles in genomic rearrangement and the provision of a stable chloroplast genome structure [41, 42]. Because of the variable copy number and the variation in length, repeat sequences have attracted considerable attention for understanding phylogenetic relationships among species, biogeography, and population genetics. A total of 210 long repeats (30–190 bp) and 382 SSRs were identified from the eight Papaveroideae chloroplast genomes (Fig. 4). *D. leptopodum* contained the highest number (50) of long repeat sequences, and *Me*. *horridula* had the lowest number (16) among the compared species. The numbers of forward and palindromic sequence repeats detected in *D. leptopodum* were 46 and 4, respectively. Similarly, forward repeats were the most frequent repeat sequences observed among the other species. These repeats exhibit a pattern similar to the patterns reported previously, and the complex repeats are pivotal components in studying the evolutionary dynamics of the chloroplast genome.

Chloroplast genome SSRs (cpSSRs) have several essential characteristics, such as abundance, maternal inheritance, and haploid nature. Based on these features, chloroplast genome SSRs are mainly used in population genetic variation and gene flow analyses [43–45] and are considered valuable markers. The significance and applicability of cpSSR markers have been reported in various other Papaveroideae species, such as using cpSSR markers to assess the population genetics of opium poppy [46]. In this study, the numbers, types, and distribution of cpSSRs were analyzed in *D. leptopodum* and related chloroplast genomes. The highest number of cpSSRs was detected in *Ma*. *cordata* (69), while the fewest cpSSRs were observed in *P. nudicaule* (20). Consistent with previous results, we identified that mononucleotide-type SSRs were the most abundant in the chloroplast genome and were biased as A and T nucleotides, resulting in an AT-rich chloroplast genome.

Increasingly, studies have shown that chloroplast genome sequences are suitable for inferring phylogenetic relationships at different taxonomic classification levels [14, 47–50]. Based on whole chloroplast genome sequences, numerous phylogenetic problems at the deep node level have been solved, for example, identifying the earliest-diverged group of angiosperms [51–53] or the phylogenetic relationships among the five tribes of Oleaceae [13]. This approach allows a better understanding of the complex evolutionary links among angiosperms. Meanwhile, the chloroplast genome dataset may also resolve shallow phylogenetic problems. Previously, Hoot et al. [54] determined the phylogenetic relationships of Papaveraceae based on the chloroplast regions *atpB*, *rbcL*, *matK*, and nuclear 26S ribosomal DNA. The results resolved the relationships of deep nodes (subfamily and tribe levels) in the family Papaveraceae but failed to resolve phylogenetic relationships at the species level using these four markers. In our study, *Dicranostigma* was sister to the tribe Chelidoniinae, with strong support obtained using the chloroplast genome (Fig. 8). Furthermore, our study revealed species clustering within *Meconopsis* and *Corydalis,* all with high bootstrap and posterior probability values.

## Conclusions

In this study, we sequenced and assembled the complete chloroplast genome of *D. leptopodum* and compared it with the chloroplast genomes of related species from the subfamily Papaveroideae. We identified important genetic resources and evolutionary dynamics of the chloroplast genome for *D. leptopodum*, such as repetitive sequences, codon usage, SSRs, sequence divergence, IR contraction and expansion, molecular evolution analyses, and phylogenetic inference. Comparative genomics indicates that the Papaveroideae chloroplast genomes are relatively conserved, with several regions of high sequence divergence identified as potential markers for phylogeny. Phylogenetic results resolved the phylogenetic position of *Dicranostigma.*

## Supplementary Information

**Additional file 1.**

**Additional file 2.**

**Additional file 3.**

## Data Availability

The complete annotated sequence of *Dicranostigma leptopodum* is deposited in the NCBI database (https://www.ncbi.nlm.nih.gov/) (GenBank accession number: OM994890). The *D. leptopodum* material was obtained from Luoyang, Henan, China, and the specimens were subsequently deposited in the Herbarium of the College of Horticulture and Plant Protection, Henan University of Science and Technology (Voucher: WL1000). These specimens were identified by Dr. Ning Wang at Henan University of Science and Technology.
